# P07 - Treatment load in the therapy management of allergic rhinitis: a UK retrospective database study

**DOI:** 10.1186/2045-7022-4-S1-P62

**Published:** 2014-02-28

**Authors:** Victoria Carter, David Price, Glenis Scadding, Shuaib Nasser, Claus Bachert, Hesham Saleh, Julian Maitland, Julie von Ziegenweidt, Dermot Ryan

**Affiliations:** 1 Research in Real Life, Cambridge, United Kingdom; 2 University of Aberdeen, Aberdeen, United Kingdom; 3 RNTNE Hospital, London, United Kingdom; 4 Cambridge University Hospitals NHS Foundation Trust, Cambridge, United Kingdom; 5 University Hospital Ghent, Ghent, Belgium; 6 Imperial College of Medicine, London, United Kingdom; 7 Meda Pharmaceuticals, London, United Kingdom; 8 Research in Real Life, Norwich, United Kingdom; 9 East Midlands Strategic Health Authority, Leicester, United Kingdom

## Background

Allergic rhinitis (AR) is poorly controlled. Treated patients, even those on multiple therapies still experience symptoms. In a UK survey, 70.5% of moderate-to-severe AR patients took ≥2 medications (either Rx or over the counter) in an attempt to achieve better and faster symptom relief.

## Aim

To explore the extent of co-prescribing by UK general practitioners (GPs) during the hay-fever season in patients with seasonal AR (SAR), perennial AR (PAR), and comorbid asthma.

## Method

A retrospective database study using the Optimum Patient Care Research Database consisting of data extracted from GP records supplemented with patient-reported outcomes from questionnaires. Patients included in the analysis had a recorded AR diagnosis and ≥1 AR therapy scripts during 1st March 2010 to 31st August 2010.

## Results

In all, 22,381 AR patients were included. Results are summarized in the table.

**Table 1:** 

1st March 2010 to 31st August 2010				
	SAR (n=16187)		PAR (n=6194)	

Therapy, n (%)	**Season Start**	**Season End**	**Season Start**	**Season End**

Single therapy	10776 (65.5)	8850 (54.7)	4764 (76.9)	2974 (48.0)

Dual therapy	3782 (23.4)	5213 (32.2)	1172 (18.9)	2445 (39.5)

Triple therapy	1615 (10.0)	2062 (12.7)	244 (3.9)	694 (11.2)

Quadruple therapy	24 (0.1)	62 (0.4)	14 (0.2)	77 (1.2)

5 therapies	0 (0.0)	0 (0.0)	0 (0.0)	4 (0.1)

**Total on combinations**	**5421 (33.5)**	**7337 (45.3)**	**1430 (23.0)**	**3220 (52.0)**

**Season End, n (%)**	**Asthma (n=8787)**		**Non Asthma (n=13594)**	
	
	SAR	PAR	SAR	PAR

**Combination therapy** Start of season End of season	1536 (24.6) 2296 (41.6)	780 (23.9) 1805 (55.2)	3885 (36.4) 5041 (47.2)	650 (22.2) 1415 (48.4)

## Conclusion

In contrast to previous surveys, these data relate to prescriptions only and show a high level of co-prescribing behavior among UK GPs. There was a significant shift to combination therapy during the season, particularly for PAR patients, with strong co-prescription evident regardless of asthma co-morbidity. These data indicate that (i) UK GPs are aware that current therapy provides insufficient symptom relief, (ii) that AR is a costly disease to treat requiring several GP visits over the season for therapy modification and (iii) there is a need for an AR therapy which provides more complete symptom relief.

